# Distinct gene expression in demyelinated white and grey matter areas of patients with multiple sclerosis

**DOI:** 10.1093/braincomms/fcac005

**Published:** 2022-01-17

**Authors:** Thecla A. van Wageningen, Emma Gerrits, Nieske Brouwer, John J. P. Brevé, Jeroen J. G. Geurts, Bart J. L. Eggen, H. W. G. M. (Erik) Boddeke, Anne-Marie van Dam

**Affiliations:** 1 Department of Anatomy & Neurosciences, MS Center Amsterdam, Amsterdam Neuroscience, Amsterdam UMC, Vrije Universiteit Amsterdam, Amsterdam, The Netherlands; 2 Department of Biomedical Sciences of Cells & Systems, Section Molecular Neurobiology, University Medical Center Groningen, Groningen, The Netherlands; 3 Center for Healthy Ageing, Department of Cellular and Molecular Medicine, University of Copenhagen, Copenhagen, Denmark

**Keywords:** multiple sclerosis, leucocortical lesions, grey matter demyelination, RNA sequencing, glial cells

## Abstract

Demyelination of the central nervous system is a prominent pathological hallmark of multiple sclerosis and affects both white and grey matter. However, demyelinated white and grey matter exhibit clear pathological differences, most notably the presence or absence of inflammation and activated glial cells in white and grey matter, respectively. In order to gain more insight into the differential pathology of demyelinated white and grey matter areas, we micro-dissected neighbouring white and grey matter demyelinated areas as well as normal-appearing matter from leucocortical lesions of human post-mortem material and used these samples for RNA sequencing. Our data show that even neighbouring demyelinated white and grey matter of the same leucocortical have a distinct gene expression profile and cellular composition. We propose that, based on their distinct expression profile, pathological processes in neighbouring white and grey matter are likely different which could have implications for the efficacy of treating grey matter lesions with current anti-inflammatory-based multiple sclerosis drugs.

## Teaser

van Wageningen *et al*. present gene expression data of demyelinated and normal-appearing white and grey matter of multiple sclerosis leucocortical lesions. They find that grey matter lesions primarily feature gene expression indicative of a microglial response while white matter lesions feature astrocytic activation and dystrophic neurons.

## Graphical Abstract

**Graphical Abstract:**
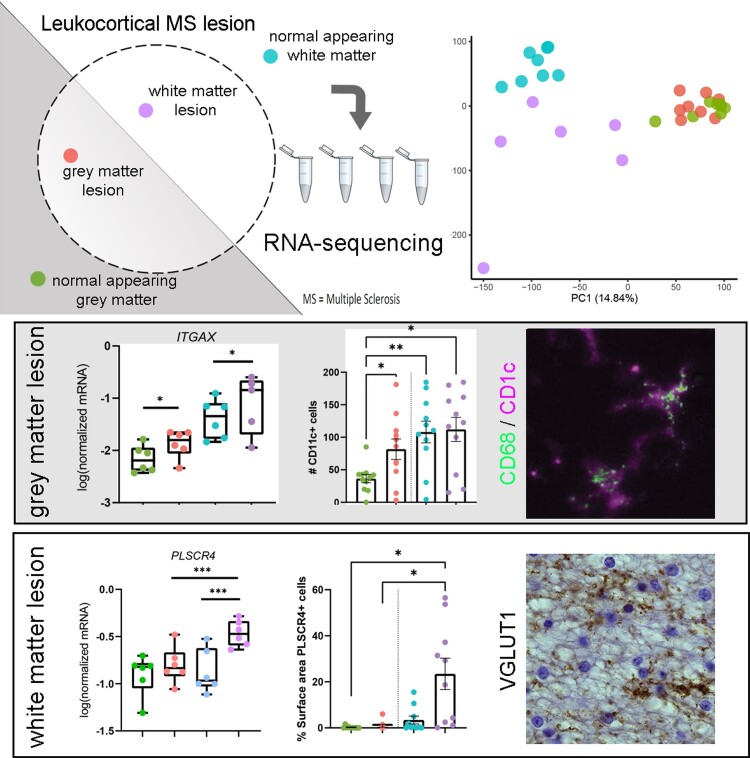

## Introduction

Multiple sclerosis is a neurological disorder pathologically characterized by inflammation, demyelination and axonal damage in the CNS. It is the most common immune-mediated neurological disease among young adults, clinically represented by heterogeneous symptoms including sensory and motor deficits as well as fatigue and cognitive dysfunction.^1^ Although demyelination is the pathological hallmark of multiple sclerosis, large differences exist between demyelinated white and demyelinated grey matter. Post-mortem analyses revealed that demyelination in the white matter is accompanied by activation of local glial cells and infiltration of peripheral leucocytes, including monocytes and lymphocytes. In contrast, demyelinated grey matter areas show a paucity of activated glial cells and little to no infiltration of peripheral leucocytes.^2–4^ Several hypotheses have been put forward as to why demyelinated white and grey matter differ in their pathology, including (i) the presence of neuronal cell bodies, suppressing an immune response in the grey matter,^5,6^ (ii) a difference in the abundance of myelin eliciting an immune response^7^ or (iii) that grey matter pathology is largely driven by the presence of meningeal infiltration of B-cells instead of parenchymal infiltration of leucocytes, as is observed in the white matter.^8–10^ Thus far, analyses of brain material from multiple sclerosis patients, suggest that, irrespective of the presence of infiltrating leucocytes, glial cell activation, chronic oxidative injury and accumulation of mitochondrial damage in axons are important contributors to the pathological processes in white matter lesions (WMLs) ultimately leading to axonal loss.^1^ Similarly, astrocyte scarring, represented by hypertrophic astrocytes and deposition of extracellular matrix proteins, is present in the demyelinated white matter only.^11,12^ In contrast, in the grey matter, the role of glial cells, including microglia and astrocytes in multiple sclerosis pathology, is largely unknown, but glial cell reactivity could be affected by CSF or meningeal-derived inflammatory factors, e.g. complement or cytokines.^13–15^ The direct relation between inflammation in meninges or CSF and cortical demyelination is, however, at least questionable.^16,17^

To date, the clear difference in pathological appearance and molecular mechanisms underlying demyelinated grey and white matter multiple sclerosis lesions have not been backed up with a more in-depth analysis that may guide understanding of pathways involved. Though there have been several studies investigating gene expression in demyelinated and normal-appearing white matter (NAWM) in humans (for a review, see Jäkel and Williams^18^) and in mouse models of multiple sclerosis (including but not limited to)^19–21^ sequencing data of multiple sclerosis (demyelinated) grey matter is relatively sparse. Until now micro-array data of multiple sclerosis normal matter^22^ have appeared. Although of interest, none of these studies looked into gene expression profile differences between grey and white matter multiple sclerosis lesions compared with normal-appearing grey matter (NAGM) and NAWM. In addition, the focus of most of these grey matter expression studies is on subpial demyelination only. We consider the comparison of gene expression profiles of pathologically confirmed demyelinated white and grey mater material compared with its respective normal-appearing matter of considerable importance. Particularly, because current drug treatment of multiple sclerosis patients focuses on reducing the inflammatory components of the disease, e.g. less influx of lymphocytes as occurs in demyelinated white matter only. In demyelinated grey matter, the relative absence of these cells may point to another, less inflammatory, mechanism underlying demyelination, which may impact upon therapy development. In the present study, we thus analysed demyelinated white and grey matter from the same histologically verified post-mortem human leucocortical multiple sclerosis lesion and normal-appearing tissue blocks by RNA sequencing to generate transcriptomic data. Using these data, we address the gene expression profile of (demyelinated) grey matter and (demyelinated) white matter and compare these to each other. In addition, we assess the effect of different cellular compositions in (demyelinated) white and grey matter and the effect on the gene expression profile and enriched pathways.

## Materials and methods

### Human brain tissue

Fresh-frozen tissue blocks and formalin-fixed paraffin embedded (FFPE) human brain tissue blocks were obtained from the Netherlands Brain Bank and the Amsterdam Multiple Sclerosis Centre, Amsterdam UMC and were selected from material available from the years 2003 to 2019. The inclusion criteria were (i) availability of both frozen and FFPE tissue, (ii) clear loss of proteolipid protein (PLP) immunoreactivity encompassing neighbouring white and grey matter, (iii) amoeboid, activated major histocompatibility complex II (MHC-II) positive cells within the demyelinated white matter of the lesion. In practice, we observed that whereas sometimes there was a leucocortical lesion present in the collected FFPE material, this was not always the case in the frozen material. Therefore, at a later time point, we also included leucocortical lesions and concomitant normal-appearing matter present in frozen or FFPE only material. In compliance with all local ethical and legal guidelines, informed consent for brain autopsy and the use of brain tissue and clinical information for scientific research were given by either the donor or the next of kin. Clinical information can be found in Table 1.

**Table 1:** Clinical data of patients of tissue used for RNA-seq, qPCR, immunohistochemistry and/or microglia tracing

#	Sex	Age	PMD	Diagnosis	Disease duration (years)	Cause of death	Tissue used for
1	f	57	7:30	SPMS	34	Euthanasia	RNA-seq
2^a^	f	47	8:35	SPMS	27	Aspiration Pneumonia	RNA-seq, qPCR
3^b^	m	56	9:35	PPMS	32	End-stage multiple sclerosis with urosepsis	RNA-seq
4^b^	f	63	10:50	SPMS	25	Infection	RNA-seq
5	f	41	8:25	SPMS	11	Natural causes	RNA-seq, IHC
6^b^	f	57	10:40	SPMS	25	Euthanasia	RNA-seq, IHC, microglia tracing
7^a^	f	54	9:25	SPMS	24	Dyspnoea followed by palliative care	RNA-seq, IHC, qPCR, microglia tracing
8	m	53	5:30	PPMS	2	Pneumonia	RNA-seq, IHC, qPCR
9	f	66	6:30	SPMS	26	Euthanasia	RNA-seq, IHC, microglia tracing
10	f	67	11:25	SPMS	28	Aspiration pneumonia	RNA-seq, IHC, qPCR, microglia tracing
11	f	66	6:00	SPMS	23	Unknown	RNA-seq, IHC
12	m	45	7:45	SPMS	20	Cardiac arrest	IHC, qPCR, microglia tracing
13	f	51	9:10	SPMS	17	Euthanasia	IHC, microglia tracing
14	f	50	10:15	SPMS	11	Euthanasia	IHC
15	m	56	6:15	PPMS	20	Pneumonia	IHC, microglia tracing
16	m	56	6:15	PPMS	20	Pneumonia	IHC, qPCR, microglia tracing
17	m	48	6:35	SPMS	16	Dehydration	IHC, qPCR, microglia tracing
18	f	74	10:40	PPMS	13	Heart failure	IHC, microglia tracing

PMD, post-mortem delay; IHC, immunohistochemistry; qPCR, quantitative PCR.

^a^ WML sample not included in RNA sequencing due to technical problems.

^b^ All samples from this patient not included in data analysis after read alignment.

### Immunohistochemistry

Fresh-frozen and FFPE tissue blocks were sectioned at 10 µm and mounted on positively charged glass slides (Menzel-gläser, ThermoFisher Scientific). Cryo-sections were fixed in 100% acetone for 45 min, dried and washed in 0.1 M Tris-buffered saline (TBS; pH 7.6), whereas FFPE sections were deparaffinized before undergoing antigen retrieval using 10 mM Tris buffer containing 1 mM ethylenediaminetetraacetic acid (Tris-EDTA, pH 9). Subsequently, all sections were incubated for 20 min with 1% H_2_O_2_ in TBS to block endogenous peroxidase, washed in TBS and incubated for 30 min in 5% donkey serum in TBS with 0.1% Triton-X (block buffer) to block non-specific antibody binding. Thereafter, sections were incubated with primary antibodies (see Supplementary Table 1) diluted in block buffer overnight at 4°C. Then, sections were washed with TBS and incubated in block buffer containing biotinylated donkey anti-mouse IgG (1:400, Jackson Laboratories), donkey anti-rabbit IgG (1:400, Jackson Laboratories) or donkey anti-goat IgG (1:400, Jackson Laboratories) or goat anti-guinea pig (1:400, Vector) at room temperature (RT) for 2 h. Subsequently, sections were washed in TBS and incubated for 1 h with horseradish peroxidase (HRP)-labelled avidin–biotin complex (ABC complex, 1:400, Vector Labs) at RT immunoreactivity was then visualized by adding 3,3-diaminobenzidine (Sigma, St Louis, USA) and sections were counterstained with haematoxylin. Sections were subsequently dehydrated in graded series of ethanol, cleared in xylene and mounted with Entellan (Merck). Slides were scanned using a Vectra Polaris slidescanner and images included in the figures were selected using QuPath.^23^

Double-labelling immunohistochemistry was performed on FFPE sections treated similar to that described above until the incubation with primary antibodies. Sections were incubated with combinations of primary antibodies diluted in block buffer, overnight at 4°C (Supplementary Table 1). Sections were washed in TBS and incubated with ImmPRESS alkaline phosphatase (AP) anti-rabbit [CD11c, phospholipid scramblases 4 (PLSCR4), excitatory amino-acid transporter 2 (EAAT2)] for 30 min at RT. Subsequently, slides were washed in TBS and incubated for 30 min at RT with Envision+ HRP labelled polymer anti-mouse [glial fibrillary acidic protein (GFAP), CD68; DAKO] or Immpress-HRP anti-goat IgG polymer detection kit [ionized calcium binding adapter molecule 1 (Iba-1), Vectorlabs]. Subsequently, AP immunoreactivity was visualized by adding Liquid Permanent Red (LPR, DAKO) and HRP immunoreactivity was visualized using the Vector SD Peroxidase kit (Vectorlab). Subsequently, sections were washed in TBS and demi-water before being dried on a heat plate at 37°C before and cleared in xylene and mounted with Entellan (Merck).

Immunohistochemically stained sections were scanned using a Vectra Polaris slide scanner or images were obtained using a Leica DM5000B microscope. Pictures of double-labelled sections were taken at wavelengths ranging from 480 to 680 nm at 63× magnification using the Nuance multispectral imaging system (PerkinElmer) and were analyzed as previously described in van Wageningen et al.^24^ In short, LPR-stained cells (red) and Vector SG-stained cells (grey) were separated based on their light emission, which yields images similar to fluorescently labelled antibodies.^25^ Using the open-source software ImageJ,^26^ compositions of the separated signals were then made to visualize co-localization (see Supplementary Figs 1 and 2).

### Laser capture microscopy

Sample preparation for laser capture microscopy (LCM) was done as previously published^27^ with slight modifications. In short: fresh-frozen tissue blocks were sectioned at 10 µm in an RNAse-free cryostat and mounted on PEN slides (Leica). Immediately after mounting, the sections were fixed in 100% molecular grade ethanol for 10 min and dried. Subsequently, the sections were stored at −80°C, paired back to back in a 50 ml tube containing silica gel as a desiccant. Upon use for LCM, sections were taken from storage and immediately put into 100% molecular grade ethanol, rehydrated to 70% ethanol and then stained in 4% cresyl violet in 70% ethanol for 45 s. After staining, sections were dehydrated in an ethanol series (up to 100% ethanol). Sections were dried and then stored again in 50 ml tubes containing silica gel on dry ice before commencing LCM. Upon use for LCM, frozen, cresyl violet-stained sections on PEN slides were transferred to 100% ethanol for 1–2 min and dried. NAWM and NAGM and demyelinated white and grey matter were localized and dissected using the Leica LMD6500 system (Leica, Germany). Per section, three squares of tissue per area were captured. Per patient, tissue was captured from 6 to 12 cresyl violet-stained sections. We repeated this procedure for subsequent quantitative polymerase chain reaction (qPCR) analysis using tissue from a subset of the same patients used for RNA-seq and two additional patients (see Table 1).

### RNA isolation and RNA integrity number determination

Pooled laser-captured samples per area per patient were collected in 50 µl extraction buffer of the ARCTURUS^®^ PicoPure^®^ RNA Isolation Kit (ThermoFisher Scientific), incubated at 42°C for 30 min (according to the manufacturer’s protocol) and stored at −80°C until further use. RNA was isolated according to the manufacturer’s protocol with an additional DNAse digestion step using the Qiagen RNAse-free DNAse kit (Qiagen, Germany). RNA quality and concentration of all samples were determined using the Agilent Bioanalyzer before and after the LCM procedure.

In total, 11 frozen tissue blocks from 11 patients with multiple sclerosis featured a Type I leucocortical lesion that fitted the previously established pathological criteria. The mean ± standard deviation (SD) RNA integrity indicated by the RNA integrity number (RIN) score of the tissue blocks before the LCM procedure was 6.2 ± 1.2. After the LCM procedure, the mean ± SD RIN value of laser-captured samples was 5.1 ± 1.2 which was considered appropriate to be used for RNA sequencing. For additional qPCR, we obtained two more frozen tissue blocks with leucocortical lesions from two additional patients. However, we could not include all the tissue blocks used for RNA-seq, a second time for qPCR as we had no left over material from some blocks. We repeated the experimental procedure to generate RNA samples for qPCR, tissue used for this experiment can be found in Table 1. More information on the RIN of these samples and the choice of housekeeping genes can be found in Supplementary Data 1.

### RNA sequencing

Sequencing libraries were prepared with the Quant Seq 3′ mRNA seq Library Prep Kit FWD (Lexogen, Vienna, Austria). The libraries were sequenced on a NextSeq platform.

### Data analysis

Quality control of the raw FASTQ files was performed with FASTQC. Bad quality bases were trimmed with TrimGalore version 0.4.5. Sequences were aligned using HiSat2 version 2.1 to the *Homo Sapiens* (GRCh38.92) reference template obtained from Ensembl and quantified with featureCounts. A quality check of aligned data was performed with FASTQC and MultiQC. Raw count matrices were loaded in R and annotated by converting the ensemble IDs to gene symbols using the corresponding gtf file. Only genes with >1 counts in at least two samples were included in the analysis.

Subsequently, data were normalized and analysed using the *EdgeR* package. A likelihood ratio test (LRT) was used to compare gene expression between the four defined groups using only tissue area as a variable (i.e. NAWM and NAGM and demyelinated white and grey matter). Subsequently, the *DEGpatterns* package was used to divide the differentially expressed genes into clusters based on gene expression pattern. The threshold for each cluster was set at a minimum of 50 genes. Up- or downregulated genes were identified with the criteria of a logFC >(−)2 and a *P*-value of <0.05. Pathway analysis of genes represented in the clusters was conducted using the *g:Profiler* package.

To determine the cell type specificity of genes, we used CIBERSORTx^28^ with single-cell data generated from frozen multiple sclerosis (demyelinated) white and grey matter by Schirmer *et al*.^22^ Data were downloaded from the Sequence Read Archives from the accession number PRJNA544731 (NCBI Bioproject ID: 544731).

### Semi-quantitative reverse transcription-polymerase chain reaction

RNA was isolated similarly to what is previously described as input for RNA-seq. For qPCR, RNA concentration and RIN values were determined using the Agilent Bioanalyzer. Per sample, 3 ng was reverse-transcribed into cDNA using the High-Capacity cDNA Reverse Transcription Kit (Applied Biosystems, Bleiswijk, The Netherlands) with random primers (50 µM, Invitrogen) according to the manufacturer's description. Semi-quantitative reverse transcription-polymerase chain reaction (RT-PCR) was performed in a total volume of 10 µl per sample consisting of 3 µl of Power SYBR Green Master Mix (Life Technologies, Carlsbad, CA, USA), with 50 µM of each forward and reverse primers (see Supplementary Table 2) and 6 ng/µl cDNA in a MicroAmp Optical 96-well Reaction Plate (Applied Biosystems, Foster City, CA, USA). The RT-PCR reaction was performed using the Quantstudio 3 PCR system (Applied Biosystems). The PCR protocol was adapted from the manufacturer’s description and featured 40 cycles with an annealing temperature of 60°C, followed by a melt curve analysis. The relative expression level of the target genes was determined by the LinReg PCR software [version 2014 4.3 (July 2014); website: http://www.hfrc.nl] using the following calculation N0 = Nq/*E*Cq (N0 = target quantity, Nq = fluorescence threshold value, *E* = mean PCR efficiency per amplicon, Cq = threshold cycle). Housekeeping genes were selected based on expression as observed in the RNA-sequencing results. *MRIP* and *SDHA* showed minimal variation between samples and between groups and thus the geomean of these two genes was used for data normalization (Supplementary Data 1). Data analysis was performed on the normalized N0 values. Primers used for qPCR can be found in Supplementary Table 2.

#### Image analysis

Per area (NAWM or NAGM and demyelinated white or grey matter) two representative images were taken of which cell counts were determined. The mean cell count per region of interest (ROI) (0.36 mm^2^) was determined. CD11c^+^ cell counts were conducted by setting a threshold using the ‘Auto Threshold’ function and subsequently counting the positive cells by using the ‘Analyze Particles’ plug-in, setting the size at 10 µm^2^. Analysis of CD11c+/Iba-1^+^ cells was conducted by first selecting Iba-1^+^ cells using the Auto-threshold function and subsequently counting the CD11c^+^ cells using the ‘Analyze Particles’ plug-in within the selected Iba-1^+^ cells. GFAP and PLSCR4 immunoreactivity were analysed by first setting a threshold using the ‘Auto Threshold’ plug-in and subsequently measuring the per cent of area stained.

#### Tracing and analysis of microglia morphology

Iba-1^+^ microglia were traced in 3D in 10 µm thick sections using a Leica DMR HC microscope coupled with Neurolucida tracing software (MBF Bioscience). The ROI was determined by the area of demyelinated grey matter and NAGM in PLP-stained sections. These ROIs were put over adjacent Iba-1 stained sections. Within these ROIs (i.e. demyelinated grey matter or NAGM), 10 microglia with a visible Iba-1^+^ cell body and haematoxylin-stained nucleus were randomly selected throughout and traced. Microglia were traced using a 63× oil objective.

Microglia tracings were analysed using the Neuronal morphology analysis package (NeuroM) for Python. Using this toolkit, the total length of all processes per cell and the total amount of forking points (i.e. a split in the process) per cell were quantified.

### Statistical analysis

Statistical analysis of all qPCR data was conducted using R using the packages *lme* to run linear mixed models and *emmeans* for *post hoc* analysis. Similar to the RNA-seq analysis, ‘tissue area’ (i.e. normal appearing or demyelinated white or grey matter) was entered as a fixed factor and ‘patient’ as a random variable. To facilitate comparisons to RNA-seq data, we report the false discovery rate (FDR). Semi-automatic quantification of immunoreactivity was analysed using repeated-measures ANOVA followed by the Tukey *post hoc* test. Analysis of microglia morphology data was assessed using linear mixed models with the patient as a random factor and lesion type area as fixed factors.

#### Data availability

RNA-sequencing data presented in this study have been deposited at https://www.ncbi.nlm.nih.gov/geo/ under the accession number: GSE149326.

## Results

### Lesion characterization before LCM

**Figure 1:**
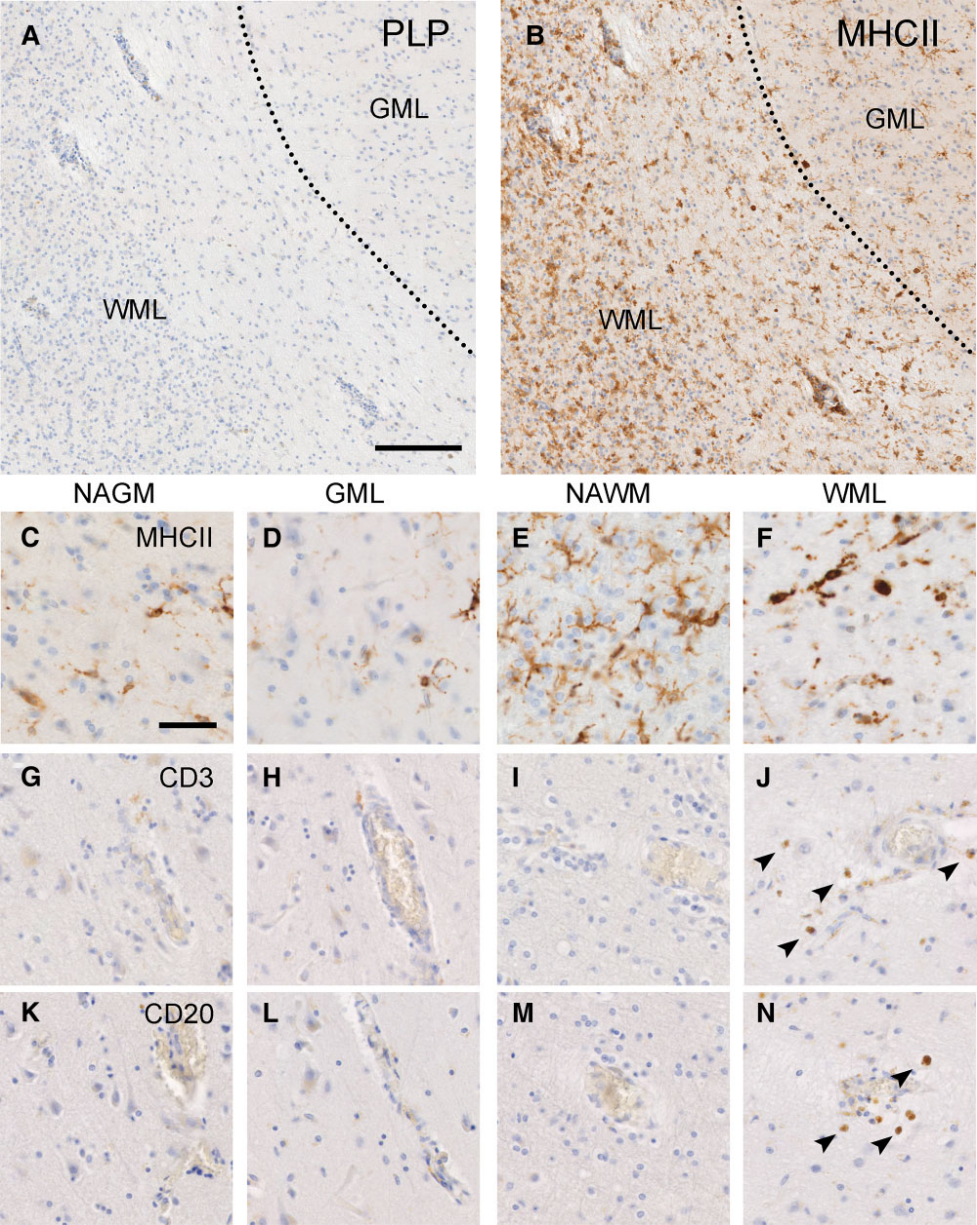
**Immunohistochemical representation of the pathology of leucocortical lesions.** Loss of myelin as shown by reduced PLP staining in GM and WM area of the lesion (**A**). Overview of MHC-II immunoreactivity in a leucocortical lesion (**B**) indicating increased MHC-II+ in the demyelinated WM which is less prominent in the demyelinated GM. Magnified overview of MHC-II immunoreactivity in the NAGM, GML, NAWM and WML (**C–F**). Overview of CD3 immunoreactivity in all leucocortical areas (**G–J**) showing infiltration of CD3 in demyelinated white matter only. Overview of CD20 immunoreactivity in all leucocortical areas (**K–N**) showing infiltration of CD20 in demyelinated white matter only. Scale bar, 500 µm (**A** and **B**) or 50 µm (**C–N**). WML, white matter lesion; GML, grey matter lesion; NAWM, normal-appearing white matter; NAGM, normal-appearing grey matter.

Lesion characterization before LCM was conducted using slides from the snap-frozen tissue blocks used for RNA sequencing. For 7 of the 11 lesions featured in the RNA-seq data, we also obtained FFPE material from the same lesions. At autopsy, the tissue block containing the Type 1 lesion was divided in half (mirrored blocks), half of the lesion was frozen and the other half fixed in formalin and paraffin embedded. When possible, lesion characterization was later verified using these FFPE tissue blocks. See Table 1 for an overview of the frozen and FFPE material used in this study. Leucocortical lesions were defined by a loss of PLP immunoreactivity indicating demyelination in grey and white matter (Fig. 1A). Leucocortical lesions are typically considered to be a type of grey matter lesion (GML) and are therefore not scored based on the presence of MHC-II^+^ cells; however, in order to facilitate the interpretation of the subsequent RNA-sequencing results from these lesions, we scored the white matter demyelinated area of the leucocortical lesion based on MHC-II^+^ as is the convention for white matter lesions (WMLs).^29,30^ Based on the presence of MHC-II^+^ cells observed in a rim surrounding the hypocellular white matter demyelinated area (Fig. 1B) and in the centre of the lesion (Fig. 1F), the presence of infiltrated CD3^+^ and CD20^+^ cells (Fig. 1I–N), we scored the white matter demyelinated areas of the leucocortical lesions as chronic active. The grey matter demyelinated area of the leucocortical lesions did not show an increase in MHC-II^+^ cells compared with the surrounding normal-appearing matter (Fig. 1B–D). Additionally, demyelinated and NAGM did not show infiltration of CD3^+^ or CD20^+^ cells (Fig. 1G, H, K and L).

### Quality control of RNA isolated from laser-captured micro-dissected tissue

**Figure 2:**
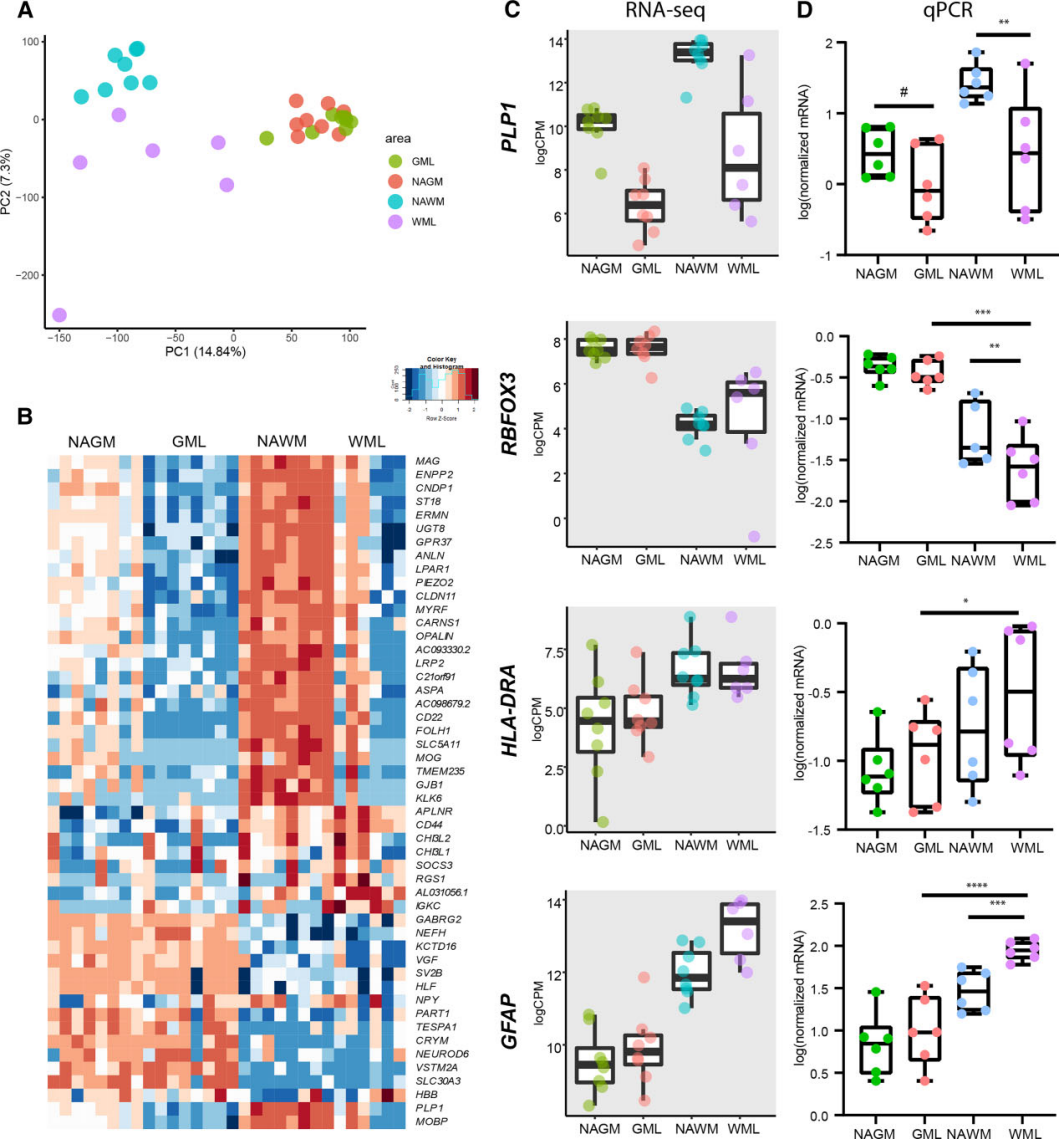
**Overall differences in gene expression between normal appearing and demyelinated white and grey matter.** PCA plot of all samples included in RNA sequencing showing separation of WM- and GM-derived samples as well as WML- and NAWM-derived samples whereas separation of GML from NAGM samples is less clear (**A**). Heatmap (log CPMs) of the top 50 genes differentiating between WML, GML, NAWM and NAGM samples according to the LRT test (**B**). Graphs of RNA-seq results indicating common genes encoding for proteins used to stage multiple sclerosis lesions (*PLP1*, *HLA-DRA*, *GFAP*) and *RBFOX3* representative of the presence of neurons in GM areas and less in WM areas (**C**). qPCR validation of RNA-seq results. *N* = 8 for NAGM, NAWM and GML samples, *N* = 6 for WML samples. *P < 0.05, **P < 0.01, ***P < 0.001, ****P < 0.0001 as shown by linear mixed models. WML, white matter lesion; GML, grey matter lesion; NAWM, normal-appearing white matter; NAGM, normal-appearing grey matter.

To verify that LCM dissected tissue from different regions as input for RNA sequencing yielded representative data, we used principal component analysis (PCA). We observed that white and grey matter samples were segregated in the first principal component (PC1) (Fig. 2A). In addition, demyelinated white matter and NAWM clearly segregated in PC2, whereas demyelinated grey matter and NAGM did not clearly segregate (Fig. 2A). This shows that gene expression differences were most pronounced between white and grey matter areas and WML and NAWM than between GML and NAGM. We identified 2352 genes which were differentially expressed (FDR < 0.05) between the four areas of the leucocortical lesions. A heatmap of the top 50 genes with the lowest FDR (Fig. 2B) showed that the four areas (demyelinated white and grey matter and NAWM and NAGM) differentiated mostly in their expression of myelin (e.g. *PLP1*, *MAG*) and oligodendrocyte-related genes (e.g. *OPALIN*, *CNP*) which were highest expressed in the NAWM followed by the NAGM and as expected showed low expression in demyelinated white and grey matter. We also observed a clear separation in the expression of neuronal-related genes (e.g. *NEFL*, *NEFH*, *GABRG2* and see Fig. 2B) which was higher in grey matter areas than white matter areas. Other genes differentiating (demyelinating) white and grey matter relate to astrocytic (*CD44*) microglial/macrophage (i.e. *CHI3L2*, *CHI3L1*) and immune responses (*IGKC*, *SOCS3*). An overview of all genes found to be differentially expressed between the four areas can be found in Supplementary Data 2. We performed qPCR to validate RNA-seq data of common markers differentiating white and grey matter [RNA Binding Fox-1 Homologue 1/3 (*RBFOX3*)], demyelinated from normal-appearing (*PLP1*) and immune activation markers (*HLA-DRA* and *GFAP*) using RNA from a repeated experiment plus two additional RNA samples (Fig. 2C and D). These data showed that also using qPCR, we observed significantly lower mRNA levels of *PLP1* [*F*(3,15) = 14.98; *P* < 0.01, Fig. 2D] indicating demyelination in samples dissected from demyelinated white matter compared with NAWM. This was also observed for demyelinated grey matter (GM) compared with NAGM, but the effect was not strongly significant (*P* = 0.077). In addition, we confirm that *RBFOX3* mRNA is significantly lower in white matter areas compared with NAGM [*F*(3,14.15) = 50.8; NAGM versus NAWM, *P* < 0.00001]. Interestingly, *RBFOX3* was also lower in demyelinated compared with NAWM (NAWM versus WML, *P* < 0.01; Fig. 2D). Though we observed no significant differences in mRNA levels of *HLA-DR* in demyelinated compared with normal-appearing areas, we did find lower mRNA levels in demyelinated grey matter than in demyelinated white matter [*F*(3,15) = 4.45; GML versus WML, *P* < 0.05; Fig. 2D]. Lastly, *GFAP* mRNA levels were higher in demyelinated white matter than NAWM (NAWM versus WML, *P* < 0.001; Fig. 2D) and *GFAP* mRNA levels were higher in demyelinated white matter compared with both grey matter areas [*F*(3,15) = 34.04; NAGM/GML versus WML, *P* < 0.0001; Fig. 2D]. Taken together, our data indicate that (i) we were able to reliably dissect areas representing normal-appearing and demyelinated white and grey matter from cresyl violet-stained leucocortical lesions and (ii) our RNA-seq data were confirmed by qPCR analysis based on relevant parameters checked.

### Demyelinated white and grey matter of leucocortical multiple sclerosis lesions has distinct gene expression profiles and cellular composition

**Figure 3:**
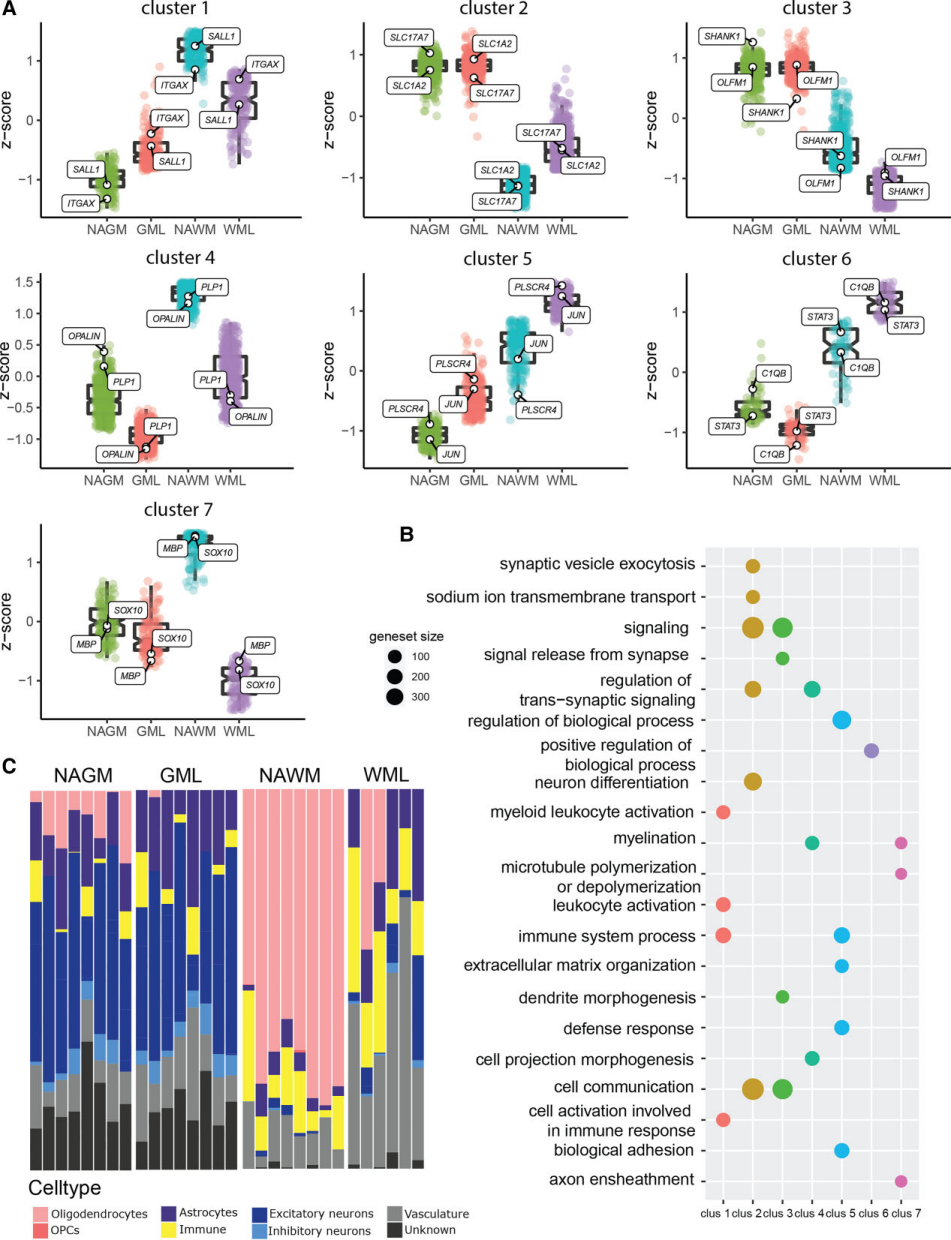
**Overview of the differences in gene expression in the four areas of a leucocortical lesion (NAGM, GML, NAWM and WML).** Cluster analysis of differential gene expression changes between leucocortical areas yields seven clusters of gene expression patterns (**A**). Pathway analysis of the seven gene expression pattern clusters (**B**). Geneset size = number of genes represented in the pathway. Proportion of genes related to different cell types as deconvoluted by CIBERSORTx (**C**). WML, white matter lesion; GML, grey matter lesion; NAWM, normal-appearing white matter; NAGM, normal-appearing grey matter.

In order to gain more insight into the differentially expressed genes between the four brain areas, we clustered the differentially expressed genes based on their expression pattern. This yielded seven clusters of gene expression (Fig. 3A). Cluster 1 showed genes with increased expression in demyelinated compared with NAGM and decreased expression in demyelinated compared with NAWM (Fig. 3A). Subsequent pathway analysis indicated that these genes are part of immune-related pathways such as ‘myeloid leucocyte activation’ and ‘cell activation involved in immune response’. Genes representative for this clusters include integrin subunit alpha X (*ITGAX*) and *SALL1* known to be expressed by microglia (Fig. 3A).^31,32^ Cluster 2 and Cluster 3 consisted of genes that were higher expressed in grey matter areas than white matter areas. These genes represent neuron (synapse)-related genes such as *SCN1A*^33^ and *NRXN1*^34^ for Cluster 2 and *OLFM1* and *SHANK1*^35,36^ for Cluster 3 (Fig. 3A). These genes are represented in pathways such as ‘signalling’ (Clusters 2 and 3), ‘signal release from synapse’ (Cluster 2) and ‘dendrite morphogenesis’ (Cluster 3, Fig. 3B). Cluster 4 and Cluster 7 represent myelin and oligodendrocyte-related genes, respectively (*PLP1*, *OPALIN*, *SOX10* and *MBP*, Fig. 3A). Pathway analysis showed enrichment for processes as ‘myelination’ (Clusters 4 and 7) and ‘axon ensheathment’ (Cluster 7, Fig. 3B). Cluster 5 consists of genes primarily higher expressed in demyelinated white matter but which also showed some elevated expression in demyelinated grey matter such as *JUN* and *PLSCR4* known to be expressed by astrocytes (Fig. 3A).^21^ Pathway analysis of these genes indicated a role for these genes in ‘immune system process’, similar to Cluster 1 but also related to ‘biological adhesion’ and ‘extracellular matrix organization’. Cluster 6 was characterized by genes higher expressed in demyelinated white matter but lower in demyelinated grey matter and features genes such as *STAT3* and *C1QB* (Fig. 3A). Pathway analysis of these genes yielded significant enrichment of only one pathway: ‘positive regulation of biological process’ (Fig. 3B). Other genes representative for each cluster and the pathways representative for each cluster can be found in Supplementary Data 3.

**Figure 4:**
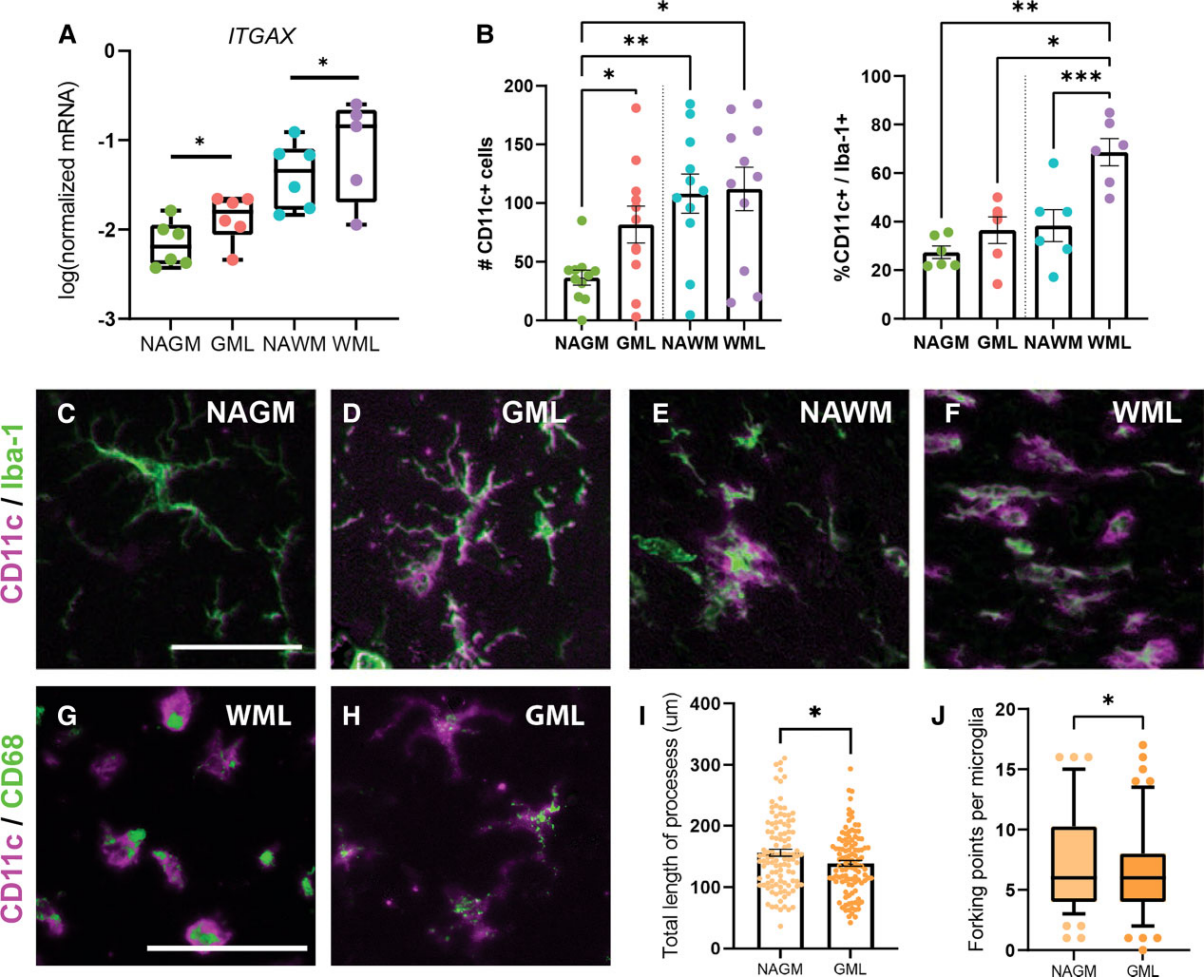
**Microglia in demyelinated grey matter (GML) of leucocortical lesions upregulate *ITGAX* and are potentially neuroprotective.** qPCR validation of RNA-seq results showing increase mRNA levels of *ITGAX* in GML versus NAGM and WML versus NAWM (**A**). Semi-automatic quantification of immunohistochemistry for CD11c (*ITGAX*) indicated increased count of CD11c^+^ cells in GMLs compared with NAGM but not WML compared with NAWM (**B**, left side). The number of Iba-1^+^/CD11c^+^ cells increased significantly in WMLs versus NAWM but not in GML versus NAGM (**B**, right side). *P < 0.05, **P < 0.01, ***P < 0.001, ****P < 0.0001, ns = non-significant as shown by linear mixed models. ROI for quantification: 0.36 mm^2^. *N* = 6 for qPCR, *N* = 11 for quantification of CD11c counts, *N* = 6 to assess co-localization of CD11c and Iba-1. Representative images of immunohistochemical stainings of CD11c (*ITGAX*) and Iba-1 showing co-localization (**C–F**). Co-localization of CD11c and CD68 indicating phagocytic activity in both amoeboid (**G**) and ramified (**H**) CD11c^+^ microglia. Microglia in GMLs of leucocortical lesions show decreased complexity as indicated by the lower total length of the processes (**I**) and less forking points (**J**). *P < 0.05 as shown by mixed models. Scale bar, 50 µm. *N* = 109 per lesion area with individually traced microglia. WML, white matter lesion; GML, grey matter lesion; NAWM, normal-appearing white matter; NAGM, normal-appearing grey matter.

Our results indicate that (demyelinated) white and grey matter have distinct gene expression profiles which may be partly due to different cellular compositions. To assess the cellular composition of the four areas represented in this study, we utilized CIBERSORTx^28^ to deconvolute our bulk RNA-seq with single-cell RNA-sequencing data derived from multiple sclerosis white and grey matter as reported by Schirmer *et al*.^22^ Deconvoluted data from CIBERSORTx confirm the loss of oligodendrocytes in demyelinated white and grey matter (Fig. 4A). Of note is that the Schirmer *et al*.^22^ data set features a relatively low number of oligodendrocyte progenitor cell (OPC) nuclei and thus there is likely an underestimation of the regulated OPC-related genes in our CIBERSORTx data. Compared with the NAWM, demyelinated white matter showed an increase in astrocyte and vasculature-related genes. This was not clearly observed in demyelinated grey matter compared with NAGM. An increase in vasculature-related genes is likely explained by an altered blood–brain barrier function in demyelinated white matter where infiltration of leucocytes is present. In line with this, we observed regulation of genes such as *ICAM2*, *CLDN5*, *JAM2* and *CDH5*, all involved in the regulation of the blood–brain barrier.^37–39^ In addition, demyelinated white matter showed a surprising increase in excitatory neuron-related genes which was not found in demyelinated grey matter (Fig. 3C). We observed a small increase in immune-related genes in both demyelinated white and grey matter compared to normal-appearing matter (Fig. 3C). Strikingly, grey matter areas feature considerable gene expression for genes classified as ‘unknown’ (Fig. 3C), highlighting the presence of genes in the (demyelinated) grey matter of which the function or cell type still needs to be elucidated.

### Microglia in demyelinated grey matter of leucocortical lesions upregulate CD11c and may be neuroprotective

To determine the validity of our RNA-seq data, we performed qPCR and immunohistochemistry for *ITGAX*, a representative gene of Cluster 1. *ITGAX* mRNA levels were indeed higher in demyelinated grey matter [*F*(3,14.01) = 24.47; NAGM versus GML, *P* < 0.05; Fig. 4A] and in the demyelinated white matter compared with normal-appearing matter (NAWM versus WML, *P* < 0.05; Fig. 4A).

Semi-automatic quantification of CD11c and Iba-1 immunoreactivity showed that the number of CD11c^+^ cells increased in demyelinated compared with NAGM [*F*(1.86,18.63) = 5.23, *P* < 0.05], but not in demyelinated compared with NAWM (Fig. 4B). Yet, the number of CD11c^+^/Iba-1^+^ cells increased significantly in the demyelinated white matter compared with NAWM only [*F*(1.48,7.38) = 17.04; *P* < 0.001, Fig. 4B] but just a tendency to increase CD11c^+^/Iba-1^+^ cells in demyelinated grey matter compared with NAGM was observed. Representative images of CD11c immunoreactivity are presented in Fig. 4C–F and Supplementary Fig. 1. As it has been reported that CD11c gene expression may be increased during demyelination and remyelination,^19^ we assessed whether CD11c^+^ cells have phagocytic properties. Indeed, we found that both amoeboid- (as observed in demyelinated white matter) and ramified-shaped CD11c^+^ microglia (as observed in demyelinated grey matter) can also express the lysosomal marker CD68, indicating lysosomal activity. We observed that microglia in demyelinated grey matter, even though they did not feature an amoeboid morphology, like microglia in demyelinated white matter, did show decreased morphological complexity compared with microglia in the NAGM as indicated by lower total length of the microglial processes [*t*_(217)_ = 2.332; *P* < 0.05; Fig. 4I] and less forking points [*t*_(217)_ = 2.214; *P* < 0.05; Fig. 4J].

### Demyelinated white matter is associated with astrocytic and neuronal changes

**Figure 5:**
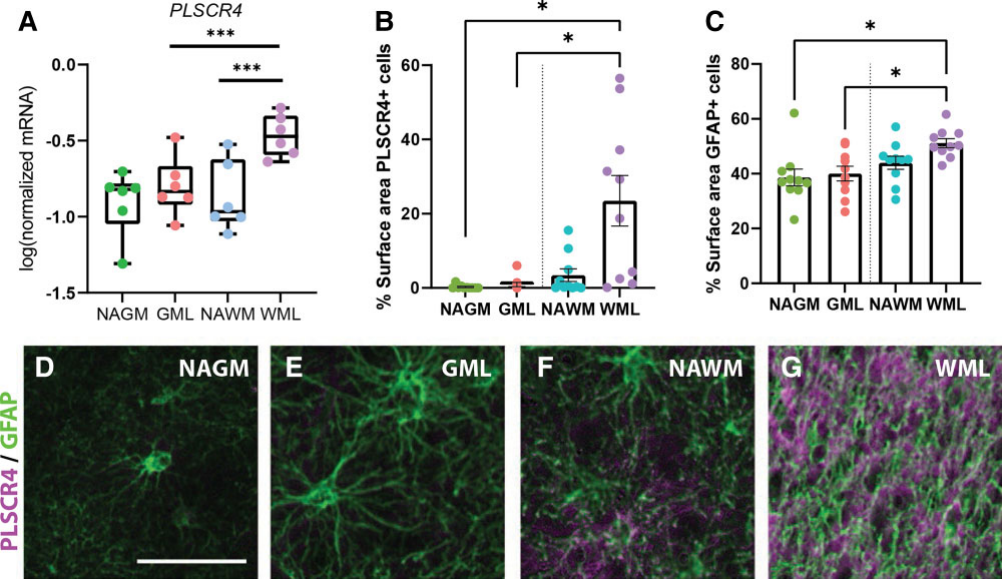
**Astrocytes in demyelinated white matter (WML) of leucocortical lesions selectively upregulate *PLSCR4***. qPCR validation of RNA-seq results showed increased mRNA levels of *PLSCR4* in WML versus GML and WML versus NAWM (**A**). Semi-automatic quantification of immunohistochemical PLSCR4 indicated increased stained surface area for PLSCR4 in WML compared with GML and NAGM (**B**). This increase was more prominent than that of GFAP stained surface area (**C**). Representative images of immunohistochemical double labelling of PLSCR4 and GFAP confirmed co-localization, particular in white matter (**D–G**). Scale bar, 50 µm. *P < 0.05, **P < 0.01, ***P < 0.001, ****P < 0.0001, ns = non-significant as shown by linear mixed models. *N* = 6 for qPCR, *N* = 10 for % surface PLSCR and GFAP. WML, white matter lesion; GML, grey matter lesion; NAWM, normal-appearing white matter; NAGM, normal-appearing grey matter.

In the demyelinated white matter, we observed changes related to the astrocytic response and neurite damage. Gene expression of *PLSCR4* showed clear regulation in the demyelinated white matter, but not in the demyelinated grey matter. Indeed, mRNA levels of *PLSCR4*, a representative gene of Cluster 5, were found to be higher expressed in the demyelinated white matter as supported by qPCR (NAWM versus WML, *P* < 0.001, NAGM/GML versus WML, *P* < 0.001; Fig. 5A). Semi-automatic quantification of the per cemt surface of immunoreactivity of PLSCR4 showed that increased PLSCR4 immunoreactivity [*F*(1.1,9.87) = 10.46] was significant in demyelinated white matter compared with NAGM (*P* < 0.05) and demyelinated white matter compared with demyelinated grey matter (*P* < 0.05, Fig. 5B). GFAP showed increased immunoreactivity [*F*(3,17) = 5.61] in demyelinated white matter compared with normal appearing (*P* < 0.05) and demyelinated grey matter (*P* < 0.05) but not NAWM (*P* = 0.16, Fig. 5C). PLSCR4 co-localized with GFAP (Fig. 5D–G) and is thus likely expressed by astrocytes. In addition, we observed a clear increase in PLSCR4 immunoreactivity in the demyelinated white matter compared with NAWM (Fig. 5F and G) and no difference in immunoreactivity between demyelinated and NAGM (Fig. 5D and E, Supplementary Fig. 2).

**Figure 6:**
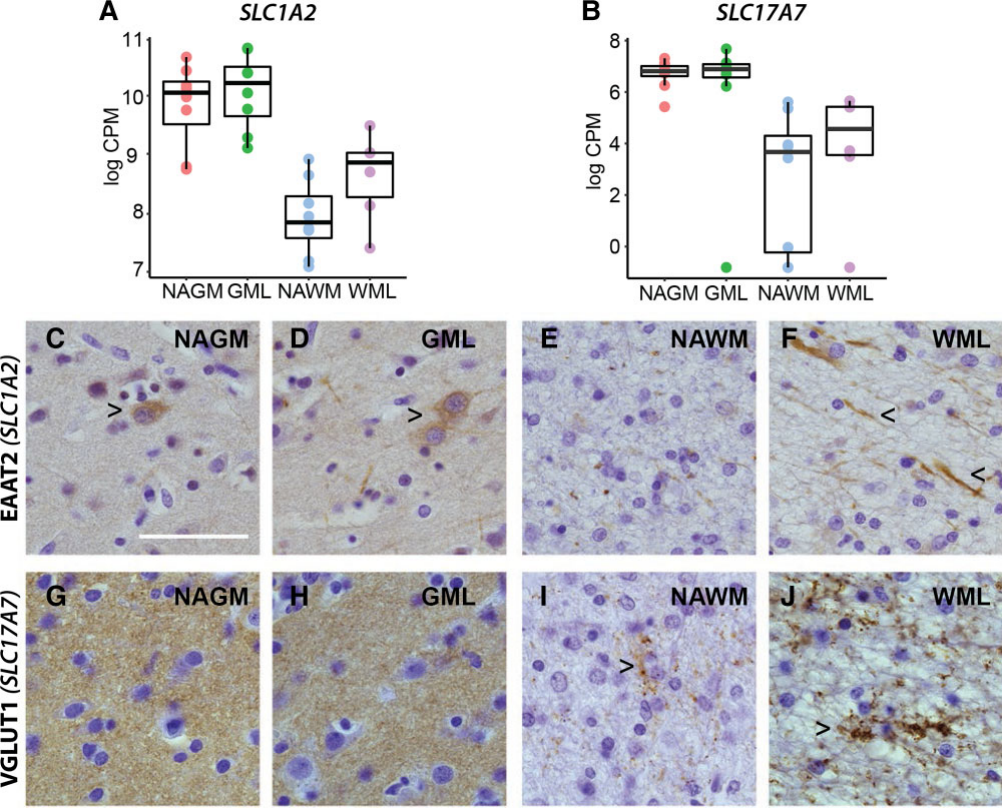
**Increased EAAT2 and VGLUT1 immunoreactivity in demyelinated white matter.** Gene expression levels of *SLC1A2* (EAAT2, **A**) and *SLC17A7* (VGLUT1) as found by RNA sequencing (**A** and **B**). Representative images for EAAT2 immunoreactivity in leucocortical lesions and normal-appearing matter (**C–F**). Right facing arrowheads indicate neuronal staining (**C** and **D**), left facing arrowheads indicate dystrophic neurites (**F**). Representative images for VGLUT1 immunoreactivity in leucocortical lesions and normal-appearing matter (**G–J**). Right facing arrowheads indicate dot-like synaptic staining in the white matter(**I** and **J**). Scale bar, 50 µm. *N* = 8 for NAGM, NAWM and GML samples, *N* = 6 for WML samples. WML, white matter lesion; GML, grey matter lesion; NAWM, normal-appearing white matter; NAGM, normal-appearing grey matter.

In addition, we observed indications of neurite damage in demyelinated white matter. EAAT2 (gene name *SLC1A2*) and vesicular glutamate transporter 1 (VGLUT1) (gene name *SLC17A7*), both representative neuron/synapse-related genes in Cluster 2 (Fig. 3A), showed higher gene expression in demyelinated white but not grey matter (Fig. 6A and B). Immunoreactivity for EAAT2 indicated some cytoplasmic neuronal staining in demyelinated and NAGM (Fig. 6C and D). A more dot-like EAAT2 immunoreactivity was observed in the NAWM (Fig. 6E), whereas in the demyelinated white matter, morphological EAAT2-positive dystrophic neurites were more apparent (Fig. 6F) as was shown before.^40^ VGLUT1 immunoreactivity was (similar to gene expression levels) high in grey matter areas, showing no difference between demyelinated and NAGM (Fig. 6G and H). In white matter areas, similar to EAAT2 immunoreactivity, a dot-like staining is observed. The amount and area of (synaptic) immunoreactive dots are increased in the demyelinated white matter, corresponding to gene expression data (Fig. 6I and J), possibly indicating increased glial/neuronal contacts.^41^ Taken together, these results suggest neurite damage present in demyelinated white but not in demyelinated grey matter.

## Discussion

In recent years, there has been a variety of studies published reporting gene expression of (laser-captured) demyelinated and normal-appearing human white matter (for a review, see Jäkel and Williams^18^) gaining insight into multiple sclerosis lesion formation in the white matter. However, it has long been known from histopathological studies that demyelinated grey matter does not feature many pathological events present in the demyelinated white matter such as infiltration of leucocytes or glial activation.^2,3^ To gain more insights in the pathology of demyelinated white and grey matter, we present gene expression of demyelinated and NAWM and NAGM areas of leucocortical multiple sclerosis lesions micro-dissected from post-mortem human brains. The strength of this study is that the experimental set-up, allowed for a direct comparison of gene expression within and between tissues taken from different patients. Generating these data was only possible by adopting a strategy where we utilized LCM to micro-dissect histologically confirmed (demyelinated) white and grey matter areas. As the micro-dissection procedure could affect the quality of the RNA used for sequencing, affecting the results, we repeated the experiment (including the addition of other patient material) by qPCR analysis as an external validation of the data. Moreover, immunohistochemical analysis was performed on FFPE mirror blocks as well as tissue from additional patients to validate certain gene data from our RNA-seq analysis at the protein level.

From these data, we find that as expected, both chronic-active demyelinated white and grey matter are defined by loss of myelin and have decreased expression of oligodendrocyte-related genes compared with NAWM and NAGM. We observed that differentially expressed genes between normal appearing and demyelinated white and grey matter could be clustered into seven clusters of gene expression patterns represented by different biological processes as shown by subsequent pathway analysis. In addition, assessment of the cellular composition of demyelinated white and grey matter and NAWM and NAGM using CIBERSORT analysis indicated differential cellular compositions between demyelinated white and grey matter areas. Taken together, these data suggest that the pathological processes underlying demyelinated white and grey matter pathology are different.

Analysis of the cluster of genes highest expressed in demyelinated grey matter indicated increased gene expression of genes involved in immune response and cellular activation. As previously mentioned, data from pathological studies have shown that a distinct pro-inflammatory response as visible in the demyelinated white matter is not present in the demyelinated grey matter. Yet, genes representative for Cluster 1 include *ITGAX* (also known as CD11c), shown by us and others to be expressed in the brain, do show increased expression in the demyelinated grey matter.^31^*ITGAX* has been shown to be upregulated in the white matter during de- but also remyelination^19^ and has been associated with phagocytic disease-associated microglia observed in, for example, Alzheimer's disease.^42^ Another gene representative for Cluster 1 is increased expression of *SALL1*, a transcriptional regulator of microglia identity.^32^ Interestingly, downregulation not upregulation of *SALL1* has been shown to lead to a pro-inflammatory microglial signature.^32^ Thus indicating that in the absence of infiltrated leucocytes (as is the case in the demyelinated grey matter), microglia in the demyelinated grey matter show a different inflammatory phenotype than in demyelinated white matter. Possibly, the increase in CD11c and CD68 in the demyelinated grey matter could indicate increased phagocytic activity and clearing of debris in these lesions, which could pave the way to more efficient remyelination and decreased inflammation which could save neurons from degeneration.^43^

Studying microglia morphology in normal-appearing and demyelinated grey matter, we observed that microglia had a more reactive phenotype, as indicated by less length and complexity of their ramifications in demyelinated compared with control grey matter. This is in contrast to what is previously reported in the cortex of multiple sclerosis patients with meningeal inflammation, where a microglia phenotype with increased complexity is observed.^15^ This could indicate that depending on the pathology present (i.e. presence or absence of meningeal inflammation) or the type of lesion studied, i.e. subpial lesions^15^ or leucocortical lesions (this study), the microglial phenotype can be different. It would be of interest for future work to compare the microglial response in GML subtypes.

We also observed two clusters of genes which were higher expressed in the demyelinated white matter and were lower expressed or showed no change in expression compared to normal-appearing matter and the demyelinated grey matter (Cluster 2 and Cluster 6). Cluster 2 is characterized by neuronal-related genes. Similar to gene expression results, overall, we observed very little differences in EAAT2 or VGLUT1 immunoreactivity in demyelinated compared with NAGM indicating that neurite damage in these areas may be less prominent. This is in agreement with data by Dutta *et al*.^44^ who also reported lack of changes in synaptic-related genes in cortical areas of multiple sclerosis patients. Though we did not quantify the immunohistochemical signal, we observed increased expression at the protein level for EAAT2 (encoded by the gene *SCL1A7*) and VGLUT1 (*SCL17A7*) in the demyelinated white matter. Expression of EAAT2 in neurons/neurites has been shown before and could possibly be indicative of a specific subtype of EAAT2 which is expressed by neurons instead of astrocytes.^45,46^ The morphology of EAAT2^+^ structures in the demyelinated white matter is representative of dystrophic neurites indicating neurite damage.^40^ In addition, increased density of VGLUT1^+^ structures likely indicate increased glial/axonal contact, possibly to support axonal function or in an effort to modulate glutamate excitotoxicity.^47,48^ Thus the altered immunoreactivity of VGLUT1/EAAT2 in the demyelinated white matter could indicate neurite transection and could explain the increase in excitatory (glutaminergic) neuronal-related genes visible in the CIBERSORTx analysis which is not prominent in the demyelinated grey matter. Of possible interest is that the different microglial inflammatory phenotype observed in the demyelinated grey matter may prevent local neurite damage. In support of this option, we found that *C1QB*, related to neuronal damage, is expressed lower in demyelinated grey matter than in the NAGM, possibly indicating some protective effect. In contrast, we observed increased gene expression of C1QB, which is related to lower VGLUT1 expression^49^ and neuronal/neurite damage in the demyelinated white matter only. Interestingly, in a recent study using single-cell sequencing of multiple sclerosis WML material, *C1QB* has been identified as a marker for ‘microglia inflamed in multiple sclerosis’.^50^ The increased expression of *C1QB* in demyelinated white matter and lowered expression in demyelinated grey matter observed in this study may imply that the classical inflamed microglial signature may not be present in the demyelinated grey matter.

Cluster 6, defined by genes higher expressed in the demyelinated white matter and lower expressed in the demyelinated grey matter also includes the transcriptional activator *STAT3*. Expression of *STAT3* is shown to be upregulated in infiltrated myeloid cells near the active demyelinating white matter.^51^ Decreased expression of *STAT3* by infiltrated myeloid cells impaired antigen presentation to CD4^+^ T cells, thereby hindering differentiation of CD4^+^ T cells to T-helper cells.^51^ Thus, increased expression of *STAT3* in demyelinated white matter could be indicative of a role for infiltrated myeloid cells, which are absent in the demyelinated grey matter which indeed shows lower expression of *STAT3*. Genes represented in another cluster of genes higher expressed in the demyelinated white matter (Cluster 5), *PLSCR4* and *JUN,* also implicate astrocyte–CD4^+^ T cells interaction in demyelinated white matter only. We observed that PLSCR4, a phospholipid scramblase,^52^ expressed by astrocytes, is selectively upregulated in the demyelinated white matter at both the gene and protein level. PLSCR4 is known to interact with CD4^+^ T cells^52^ In addition, increased gene expression of *JUN*, which has been described to be expressed by a cluster of astrocytes propagating disease which is driven by MAF BZIP Transcription Factor G (MAFG) which in turn is induced by GM-CSF released by T cells, also suggest astrocyte T-cell interactions in the demyelinated white matter only.^21^ It is postulated that the astrocytic reactivity as defined by a hypertrophic morphology could be driven by the interaction of astrocytes with T cells as it has been shown that astrocytes extend their processes towards T cells *in vitro*.^53^ This increase in astrocyte reactivity could lead to increased extracellular matrix deposition,^11^ as is indicated by the pathway analysis, which could modify the lesion environment and hinderj remyelination.^12^

Taken together, our study highlights distinct gene expression profiles of demyelinated white and grey matter areas in multiple sclerosis, within the same leucocortical lesion. The here observed limited overlap in gene expression between demyelinated white and grey matter can have potential consequences for the understanding of local ongoing pathological processes and identifying subsequent targets for the development of treatment for multiple sclerosis. Currently, treatment development and efficacy are mostly based on inflammatory demyelinated white matter lesions, as these are readily visible on conventional MRI, whereas demyelinated grey matter is not. Though we find evidence for inflammatory activity in both demyelinated white and grey matter, the inflammatory profile in these two lesion types is very different. Microglia in the demyelinated grey matter may adopt a more anti-inflammatory signature after demyelination in the absence of infiltrated leucocytes, whereas in the demyelinated white matter, inflammation may be propagated by astrocyte T-cell interactions^21^ notably absent in the demyelinated grey matter. In addition, in more inflammatory demyelinated white matter, we observed clear neurite damage, which we did not observe in the neighbouring grey matter. This could implicate that neurite damage (as observed by increased *EAAT2*) and subsequent alterations in excitatory synapses are mediated by inflammation through activated microglia as proposed before^54^ and therefore not present in the demyelinated grey matter samples used for this study which featured possibly a different type of inflammatory microglia. Though our data indicate neuroprotective events present in demyelinated grey matter of leucocortical lesions, grey matter demyelination or neuronal degradation may ultimately be responsible for the silent disease progression observed in long-term multiple sclerosis patients.^55,56^ Moreover, multiple sclerosis patients that present with a cortical first phenotype (meaning they present early on with cortical atrophy) are less likely to respond to treatment,^56^ possibly as grey matter pathology is less associated with overt classical inflammation. Further study of pathological and cellular events in the (demyelinated) grey matter could provide more insights in multiple sclerosis disease pathology. For example, in this study, we focused on demyelinated grey matter of leucocortical areas whereas much of the current literature focuses more on subpial demyelination (with or without meningeal inflammation). More research is needed into comparing pathological events in different GMLs as these may have different origins.^17^ In this respect, we propose that targeting white or grey matter-specific multiple sclerosis pathology-associated genes could possibly be more beneficial for the treatment of progressive multiple sclerosis when grey matter demyelination becomes more prominent.

## Supplementary Material

fcac005_Supplementary_Data

## Abbreviations

CD =: cluster of differentiation
EAAT2 =: excitatory amino-acid transporter 2
FDR =: false discovery rate
FFPE =: formalin-fixed paraffin embedded
GFAP =: glial fibrillary acidic protein
GM =: grey matter
GML =: grey matter lesion
HRP =: horseradish peroxidase
Iba-1 =: ionized calcium binding adapter molecule 1
ITGAX =: integrin subunit alpha X
LCM =: laser capture microscopy
LPR =: liquid permanent red
MHC-II =: major histocompatibility complex II
NAGM =: normal-appearing grey matter
NAWM =: normal-appearing white matter
PLP1 =: proteolipid protein 1
PLSCR4 =: phospholipid scramblases 4
RBFOX3 =: RNA Binding Fox-1 Homologue 1/3
RIN =: RNA integrity number
ROI =: region of interest
TBS =: Tris-buffered saline
VGLUT1 =: vesicular glutamate transporter 1
WM =: white matter
WML =: white matter lesion
PCA =: principal component analysis
