# Supplementary material for: Distinct gene expression in demyelinated white and grey matter areas of patients with multiple sclerosis
Source: Brain Commun. 2022 Jan 17;4(2):fcac005. doi: 10.1093/braincomms/fcac005 (PMC8914505; doi:10.1093/braincomms/fcac005)
Supplement: fcac005_Supplementary_Data [file fcac005_supplementary_data.zip › Supplementary tables and figs.pdf]

Supplementary Tables

Supplementary Table 1: Primary antibodies used for immunohistochemistry

**Primary antibody**	**Target**	**Ab Dilution**	**Antigen Retrieval**	**Source (article number)**
Rabbit anti CD11c	Cluster of differentiation 11c	1:250	Tris/EDTA pH 9	Abcam, U.K. (ab52632)
Mouse anti MHC-II (HLA-DR)	Major histocompatibility complex II	1:1000	Tris/EDTA pH 9	Clone LN3, Pierce, ThermoFisher (MA5-11966)
Mouse anti PLP	Proteolipid protein	1:250	Tris/EDTA pH 9	Serotec (MCA839G)
Goat anti IBA1	Ionized calcium-binding adapter molecule 1	1:500	Tris/EDTA pH 9	Abcam, U.K. (ab5076)
Mouse anti GFAP	Glial fibrillary acidic protein	1:1000	Tris/EDTA pH 9	Sigma (G3893)
Rabbit anti PLSCR4	Phospholipid scramblase 4	1:1000	Citrate pH 6	ThermoScientific (PA5-51697)
Rabbit anti CD3	Cluster of differentiation 3	1:200	Citrate pH 6	DAKO (A0452)
Mouse anti CD20cy	Cluster of differentiation 20	1:200	Tris/EDTA pH 9	DAKO (M0755)
Mouse anti CD68	Cluster of differentiation 68	1:500	Tris/EDTA pH 9	DAKO (M0814)
Mouse x EAAT2	Excitatory amino-acid transporter 2	1:100	Citrate pH 6	Abcam (ab77039)
Guinea pig x VGLUT1	Vesicular glutamate transporter 1	1:2000	Tris/EDTA pH 9	Synaptic systems (135304)

Ab=antibody

Supplementary Table 2: Primers used for qPCR

**Gene name**	**Forward primer (5'- 3')**	**Reverse primer (3'- 5 ')**
RBFOX3	CAGCGACAGTTACGGCAGA	GAGAAGGAAACGGTGGAAGGT
HLA-DRA	AGCTGTGGACAAAGCCAACCTG	CTCTCAGTTCCACAGGGCTGTT
GFAP	GCAGATTCGAGAAACCAGCC	GCTCCTGCTTGGACTCCTTA
PLP1	GCAAGACCTCTGCCAGTATAGG	GGACAGAAGGTTGGAGCCACAA
ITGAX	GATGCTCAGAGATACTTCACGGC	CCACACCATCACTTCTGCGTTC
AIF1	CCCTCCAAACTGGAAGGCTTCA	CTTTAGCTCTAGGTGAGTCTTGG
PLSCR4	CCTTCAGATGCACCTGCTGTTG	CCGCAACAAAGCCAATGGTGAC
*MRIP**	CGGGTAAAGGAATCGGAAATAC	GTACTTCTTGTCCCGCAGTG
*SDHA**	CCAGGGAAGACTACAAGGTGCGGA	AGGGTGTGCTTCCTCCAGTGCT

** = used as housekeeping gene*

Supplementary Figures

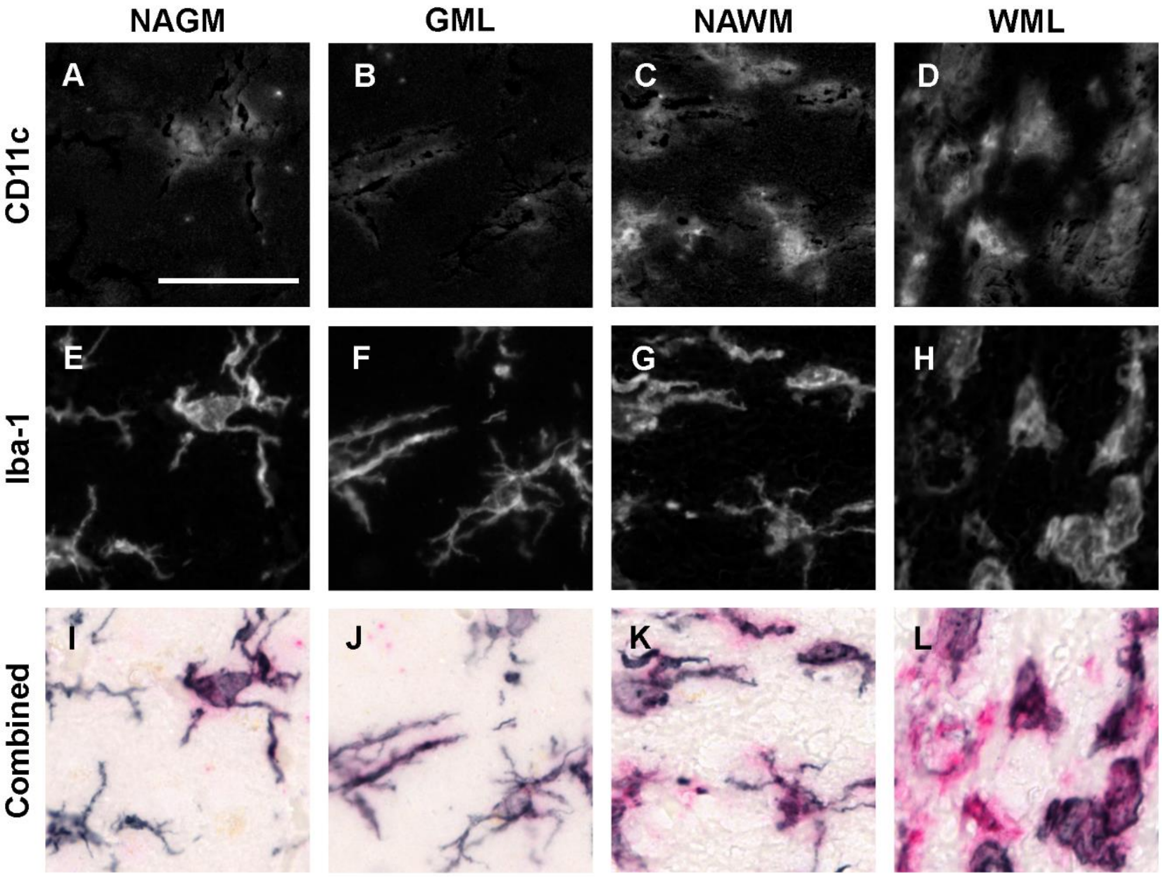

Supplementary figure 1: Representative images of extracted signals from the Nuance Spectral imaging system for CD11c (A-D), Iba-1 (E-H) and the original brightfield double-labeled images showing the combined signals (I-L). Scalebar = 50 µm. NAGM = normal appearing grey matter, GML = grey matter lesion, NAWM = normal appearing white matter, WML = white matter lesion.

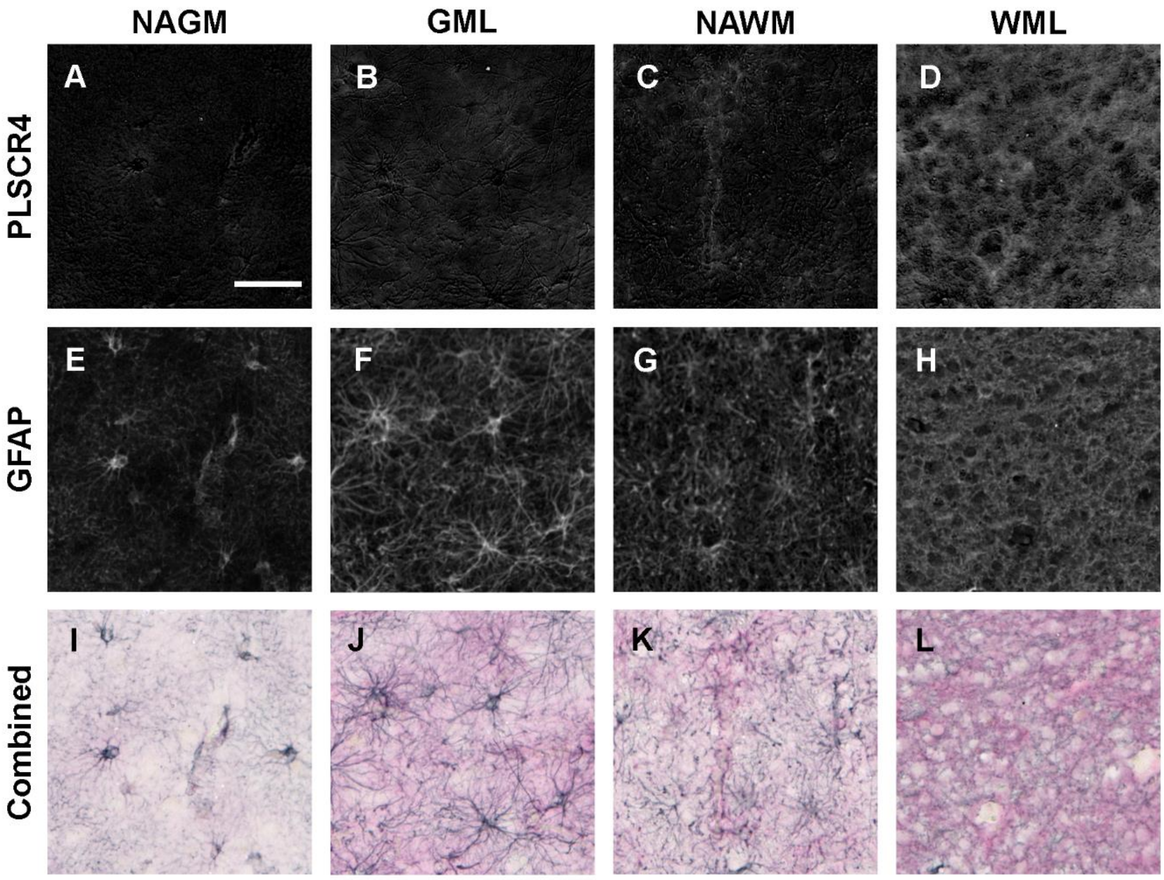

Supplementary figure 2: Representative images of extracted signals from the Nuance Spectral imaging system for PLSCR4 (A-D), GFAP (E-H) and the original brightfield double-labeled images showing the combined signals (I-L). Scalebar = 50 µm. NAGM = normal appearing grey matter, GML = grey matter lesion, NAWM = normal appearing white matter, WML = white matter lesion.
