# Supplementary material for: Distinct gene expression in demyelinated white and grey matter areas of patients with multiple sclerosis
Source: Brain Commun. 2022 Jan 17;4(2):fcac005. doi: 10.1093/braincomms/fcac005 (PMC8914505; doi:10.1093/braincomms/fcac005)
Supplement: fcac005_Supplementary_Data [file fcac005_supplementary_data.zip › Supplementary_Data_1.docx]

Supplementary information RIN value tissue blocks

The RNA integrity (indicated by the RIN value) of the tissue blocks before commencing with the LCM procedure was 6.2 ± 1.2 (mean ± SD). After the LCM procedure, the RIN values decreased slightly to 4.6 ± 1.5. After read alignment of the RNA-seq data obtained from LCM material, data from three patients showed deviating alignment metrics compared to the other samples, therefore all RNA sequencing data from these patients were excluded from the analysis.

Tissue blocks of two of the excluded patients had a low RIN value (<4) already before the LCM procedure (patient #2 and #3, see table 1). whereas the tissue block of the third excluded patient had an acceptable RIN value of 6.5 (patient #5, see table 1). Still, the RIN values of the tissue blocks of the 8 patients used for subsequent data-analysis was 6.9±1.2 before and 5.1± 1.2 after the LCM procedure which was considered of enough quality, according to the RNA-sequencing kit, to perform RNA-seq on.

For the qPCR analysis, we repeated the whole experiment, again (with the addition of two more tissue blocks from different patients) gathering tissue using LCM and subsequently determining the RIN values. This time, the average RIN values used for qCPR were lower (3.4 ±0.74), likely because part of the tissue had already been handled and used before for RNA-seq analysis. When using degraded samples, correct choice of housekeeping genes is essential^2^. We thus utilized our previously generated RNA-seq data to select two housekeeping genes which showed minimal variation between the samples, especially between samples taken from white and grey matter areas or between normal appearing and demyelinated areas (see Supplementary Figure 3). In addition, we observed that we obtained better qPCR curves when using cDNA generated using random primers instead of Olig-DT primers (unpublished data).


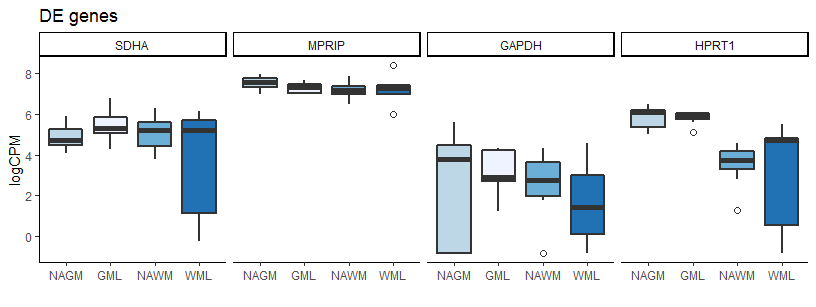


**Supplementary Figure 3**: Graphs of housekeeping genes selected for normalization (*SDHA* and *MPRIP*) and two examples of housekeeping genes which showed too much variation between samples (*GAPDH* and *HPRT1*). As there is still some variation present between groups for expression of *SDHA* and *MPRIP*, we used the averaged expression of both genes for normalization.
